# Clinical Features and Risk Factors of Mortality in Patients with Posterior Reversible Encephalopathy Syndrome

**DOI:** 10.1155/2022/9401661

**Published:** 2022-11-14

**Authors:** Hong-Wei Cui, Ru-Yi Lei, Bo-Ai Zhang

**Affiliations:** ^1^Department of General ICU, The First Affiliated Hospital of Zhengzhou University, Zhengzhou, China; ^2^Department of Emergency ICU, The First Affiliated Hospital of Zhengzhou University, Zhengzhou, China; ^3^Department of Neurology, The First Affiliated Hospital of Zhengzhou University, Zhengzhou, China

## Abstract

**Objective:**

Although the prognosis of posterior reversible encephalopathy syndrome (PRES) is usually favourable and most patients wholly recover, the disorder can result in death in some patients. To date, the data on clinical features and risk factors for death are still lacking; therefore, we aim to investigate the clinical features and long-term prognostic risk factors of PRES in the present study.

**Methods:**

The patients with PRES were identified from the First Affiliated Hospital of Zhengzhou University from June 2011 to June 2020. Clinical characteristics, laboratory tests, magnetic resonance imaging examinations, and treatment of all patients were analyzed retrospectively. All patients were followed up by telephone. Finally, the patients were divided into the survival group and death group for prognosis analysis.

**Results:**

A total of 92 patients with PRES were included; 84.8% of whom were female, with an average age of 25.4 (5–66) years at the onset of PRES. Epilepsy was the main clinical manifestation (72.8%). The in-hospital mortality rate was 2.17%. The 3-year all-cause survival rate for PRES patients was 86%. In univariate analysis, patients with systemic lupus erythematosus (*P* = 0.027) and blood transfusion history within 1 month before onset (*P* = 0.027), need for dialysis (*P* ≤ 0.001), nephritis (*P* = 0.010), stroke (*P* = 0.016), and heart failure (*P* = 0.016) were associated with death. In multivariate analysis, we found that heart failure (OR = 0.095, 95% CI 0.020 to 0.441) and stroke (OR = 0.033, 95% CI 0.002 to 0.467) were independent risk factors for death in PRES patients, while pregnancy was a protective factor for death in PRES patients (OR = 7.978, 95% CI 1.446 to 44.006).

**Conclusions:**

Our results indicate that PRES could be considered as a sign of a very high-risk patient. We also demonstrated that heart failure and stroke were independent risk factors for death in patients with PRES; moreover, pregnancy was a protective factor.

## 1. Introduction

Posterior reversible encephalopathy syndrome (PRES) is a clinical radiological syndrome first described by Hinchey et al. in 1996 [1]. The pathogenesis of PRES remains unclear. Headache, epilepsy, and visual disturbance are the main clinical manifestations. The typical magnetic resonance imaging (MRI) findings are bilateral asymmetric isodensities or low-density T1 signals and high T2 and fluid-attenuated inversion recovery (FLAIR) signals in the temporo-parieto-occipital lobe region, which are consistent with vasogenic edema [1, 2].

With the development of imaging technology, more and more cases have been reported and it has been reported that PRES is associated with a variety of disease states such as hypertension, kidney disease, autoimmune disease, and cytotoxic drug therapy [1, 2]. Fortunately, the disease is generally considered reversible; nevertheless, if not promptly diagnosed and treated, it may lead to death or irreversible neurological defects. Some studies have found that the follow-up mortality rate within 1–3 months is 3–6% [3, 4], so early and timely diagnosis is particularly important.

There is currently no research on the risk factors of long-term death in PRES, but if the risk factors that affect the prognosis can be clarified, it may be of great help to guide treatment. Therefore, for the first time, this article used a large-sample retrospective cohort study to understand the clinical characteristics of PRES and determine the long-term death risk factors, so that the risk factors can be diagnosed and effectively intervened to improve the prognosis.

## 2. Materials and Method

We retrieved all patients with a clearly diagnosed PRES who were hospitalized in the First Affiliated Hospital of Zhengzhou University from January 2011 to June 2020 in the electronic medical record system. Patients met the following criteria: (1) Acute neurological symptoms include headache, encephalopathy, epilepsy, visual impairment, or focal defects; (2) magnetic resonance suggests angiogenic edema (T2) and magnetic resonance FLAIR with high signal, apparent dispersion coefficient (ADC) with high signal, and diffusion weighted magnetic resonance imaging (DWI) with low signal, etc.; (3) clinical or radiological findings are reversible; and (4) other white matter lesions were excluded. Typical MRI manifestations are bilateral asymmetric occipital lobe lesions, and other imaging manifestations are atypical lesions [1, 2]. Patient clinical characteristics, laboratory data, and treatment history were recorded in detail. Follow-up of the patient's survival is done by telephone. Patients who were lost to follow-up were excluded. Finally, we divided all patients into the survival group and death group for statistical analysis. An expert neuroradiologist reviewed MRI images of all cases.

### 2.1. Statistical Analysis

Continuous data are expressed as mean ± standard deviation, and differences are analyzed by Student's *t*-test. The categorical data is expressed by the rate, and the *χ*2 test is used to compare and analyze the categorical data. Significant and clinically significant variables of univariate analysis were included in the analysis. We used logistic regression analysis to find risk factors for death in PRES, and the results were expressed by the OR value and CI. A *P* value less than 0.05 is considered statistically significant, and the research data was analyzed by SPSS 25.0 software. The Kaplan-Meier method was used to describe the survival rate.

## 3. Result

A total of 102 patients with PRES were found; finally, 92 patients were included in the study, including 35 systemic lupus erythematosus (SLE) patients, 42 pregnant patients, 8 SLE concurrent pregnant patients, 4 patients after stem cell transplantation, 4 patients undergoing recent surgery, 3 patients with nephrotic syndrome, 2 cases with hemophagocytic syndrome, 1 case of Sjogren's syndrome, 1 case of polycystic kidney disease, 1 case of adrenal adenoma, and other patients with unknown primary diseases. Fifty-six patients were admitted to the hospital with clinical symptoms of PRES, and the rest developed PRES during admission. During the entire treatment process, 46 patients were admitted to the intensive care unit for treatment.

### 3.1. Basic Features

The basic characteristics are summarized in Table 1. 78 patients were women, and the male to female incidence ratio was 1 : 5.57. There was no statistical difference between the two groups. The median age of onset of PRES was 25 (5–66) years, of which 73.9% of patients were under 30 years of age. Only 19.6% of the patients had a long-term history of hypertension before the onset, while up to 83.7% of the patients had hypertension at the onset. There was no significant difference in the hypertension values between the two groups at the onset. 38% of PRES patients had SLE, and the incidence of SLE was higher in the death group (62.5% vs 32.9% *P* = 0.027). There was a higher proportion of blood transfusion history in the death group within 1 month before the onset (31.3% vs 6.6% *P* = 0.027). Pregnancy in the survival group was more common (55.3% vs 12.5% *P* = 0.002).

### 3.2. Clinical and Laboratory Performance

The clinical manifestations are summarized in Table 2. Epilepsy was the main clinical manifestation of PRES. Up to 72.8% of patients had epilepsy. Headache, visual impairment, and coma were 53.3%, 31.5%, and 18.5%, respectively, and there was no statistical difference in clinical symptoms between the two groups. Renal involvement was more common in the death group (75% vs 39.5% *P* = 0.010), and more patients in the death group received dialysis before PRES (50% vs 7.9% *P* ≤ 0.001). We found that heart failure was more common in the death group (37.5% vs 6.6% *P* = 0.001), and the incidence of stroke was also higher than that in the survival group (18.8% vs 1.3% *P* = 0.016).

The median duration of SLE before the occurrence of PRES was 29 (0.25–156) months. PRES occurred in 7 patients at the first hospitalization, and 1 patient was admitted to the hospital with PRES symptoms as the first manifestation. 91.4% of patients had lupus nephritis, and about 34.4% of them required blood purification treatment. When PRES occurred, the Systemic Lupus Erythematosus Disease Activity Index (SLEDAI) score could reach an average of 26.3, suggesting that all SLE patients were highly active.

PRES occurred when the median age of pregnant women was 27 (17–39) years. Thirty-one cases (70.5%) developed neurological symptoms after delivery, of which 70.9% (22/31) underwent cesarean section. The median time for PRES to appear after cesarean section was about 5.5 (1–40) days. One case of PRES resulted in stillbirth.

Consistent with clinical manifestations, patients in the death group had higher creatinine and urea nitrogen but the laboratory findings of the two groups were not statistically significant. The laboratory data is summarized in Table 3.

### 3.3. Imaging Performance

The magnetic resonance performance is summarized in Table 4. All patients underwent emergency cranial magnetic resonance examination, all patients involved subcortical white matter, and only 16 (17.4%) patients had cortical involvement. 74 (80.4%) patients showed typical PRES performance, and there was no statistical difference between the two groups (87.5% vs 78.9% *P* = 0.433), of which 1 patient had only right parietal lesions at the beginning; however, typical lesions appeared after the condition worsened. In addition to common lesions in the temporoparietal occipital region, the frontal hemisphere of the brain was also common; as many as 72 (78.3%) patients had frontal lobe involvement, including 58 cases with bilateral involvement. The cerebellar hemisphere was also involved frequently (26/92), of which 73.1% (19) cases were bilateral. Followed by lesions in the basal ganglia, 64.7% (11/17) of the patients were bilateral. There were 12 cases of lateral ventricle and corpus callosum. There were 10 cases of thalamus, while brain stem disease was rarer (7 cases). ADC sequences of all patients showed hyperintensity. Up to 63 cases showed hyperintensity on DWI. DWI hyperintensity was mainly located in the small lesions at the site of large angioedema, which mainly appeared in patients with whole brain involvement on imaging. In previous studies, DWI hyperintensity may be related to poor prognosis [5] but there was no statistical difference between the two groups in this article (87.5% vs 64.5% *P* = 0.072). 7 patients underwent enhanced magnetic resonance, and 3 showed patchy enhancement. Stroke occurred in 4.3% (4/92) of patients, including 2 cases of acute cerebral infarction, which occurred when PRES lesions were significantly improved, and the other 2 cases had subarachnoid hemorrhage. Thirty-four patients underwent MRI scans again. The median time for MRI lesions to improve or disappear was 9 (3–60) days. The lesions disappeared completely at the shortest 7-day reexamination and improved after the longest 60-day reexamination. The reexamination revealed that 2 (5.9%) patients had no improvement in MRI. One case even had acute cerebral infarction at 2 weeks, and the lesion in another case did not disappear in the 140-day reexamination.

### 3.4. Treatment and Prognosis

The prognosis of treatment is summarized in Table 5. During the entire course of the disease, 7 patients (7.6%) required tracheal intubation for mechanical ventilation and there was no statistical difference in the intubation rate between the two groups. Patients with epilepsy as clinical manifestations were treated with antiepileptic drugs in emergency at that time, and 53.7% (36/67) patients needed oral antiepileptic drugs after seizure is being controlled. Patients with hypertension after the onset required an average of 2.56 kinds of antihypertensive drugs to fully control their blood pressure.

For SLE patients with PRES, the average dose of glucocorticoids before the onset was 46.1 mg/d based on the equivalent dose of methylprednisolone and the glucocorticoid dose after the onset increased significantly (*P* < 0.05) and the average doses reached 87.2 mg/d, which is associated with high SLE activity.

Pregnancy was terminated in 13 patients when neurological symptoms developed, 11 of which required emergency cesarean section. The median week of termination of pregnancy was 34 (25–37) weeks, and symptoms improved quickly after termination of pregnancy. The risk of pregnancy-related death was the lowest, but it had a greater impact on the prognosis of the fetus, leading 1 miscarriage in 1 patient and stillbirth in 1 patient.

After treatment, patients with PRES needed an average hospital stay of 19 days and the death group needed longer but there was no significant statistical significance. 59.8% (55/92) patients recovered completely within 72 hours, and 90% patients recovered within a week, which is different from foreign reports that 78.3% of patients recovered within 24 hours.

The median follow-up time of 92 PRES patients was 33 (0.25–95) months, and 16 patients died (Table 6), of which 8 patients died of the progression of the primary disease itself, 5 patients died of lung infection, 2 patients died of heart failure, and 1 patient died of another operation. Up to 62.5% (10/16) of the patients died within half a year of the onset, of which 2 died during hospitalization, and the in-hospital mortality rate was 2.17%. The other 6 cases died 9–53 months after the onset. The Kaplan-Meier survival curve analysis showed that the 3-year all-cause survival rate of PRES patients was 86%.

### 3.5. Risk Factors Related to PRES

After multivariate analysis (Table 7), we found that heart failure and stroke were independent risk factors for long-term death in PRES. This article also found that pregnancy was a protective factor for long-term death in PRES.

## 4. Discussion

Since Hinchey et al. [1] first described PRES in 1996, the number of cases reported has gradually increased with the development of imaging technology. Previous studies have shown that SLE, kidney disease, autoimmune disease, preeclampsia/eclampsia, cytotoxic drug treatment, and other disease states are related with the occurrence of PRES [1–6]. Although PRES is mostly reversible, serious complications such as ischemia, bleeding, and death still occur [7, 8]. At present, there is still a lack of large-scale clinical data on PRES, especially the study on long-term prognosis and risk factors. However, this information may be of great value for clinicians to diagnose in time and to guide the prevention of risk factors to improve the long-term prognosis. Therefore, in this article, we aim to explore the clinical features and determine the death risk factors for PRES through the large-sample retrospective clinical controlled study.

Similar to previous studies, most of the patients were young women [1–8], which is related to the specific disease states that PRES occurs in. This article found that pregnancy and SLE accounted for the first and second causes of PRES, respectively, 47.8% and 38.0%, which explains the age and gender characteristics of PRES. However, in another large study on infants and young PRES patients [9], neoplastic diseases and blood diseases were the top two causes of PRES, while the blood diseases accounted for only 4.4% and only 1 case (1.1%) of neoplastic diseases in this paper. It may be the difference between adult and younger PRES patients, but it may also because of the lack of awareness of children's PRES.

In present study, PRES occurred more frequently in young women but there was no statistical difference in age and gender between the two groups. However, pregnancy and SLE, which were the first two causes of PRES, had significant statistical differences between the two groups and the incidence of SLE was higher in the death group, which confirmed our previous study once again that PRES is a sign of poor prognosis for SLE [10]. The mortality of SLE with PRES in this article was 28.6%, which is similar to the other two studies, 29.17% and 26.92% [11, 12]. The high mortality rate is related to the higher activity of SLE. The average SLEDAI score could reach 26.3, and hormones were needed to increase to control the symptoms after the onset of PRES, which also support the above statement that the high activity of SLE is related to mortality, mainly because the high activity of SLE can lead to multiple organ diseases and increase the mortality rate. Previous literature reported that the incidence of PRES in pregnant patients can reach 0.22% [6]; this article found that pregnancy was the first cause of PRES, which is consistent with it. The proportion of pregnant patients in the death group was significantly less than that in the survival group; moreover, we confirmed that pregnancy was a protective factor for the death of PRES in multivariate analysis, showing that PRES in pregnant patients has the characteristics of high morbidity and low mortality, which is consistent with previous studies [5, 6, 8]. However, the specific reason is not still clear. Chen et al. believed that this may be because pregnancy-related PRES is a relatively benign pathophysiological mechanism, and perinatal women can be carefully monitored for vital signs [5]. Pande et al. found that all patients had completely reversible lesions in a report on pregnancy relevant PRES studies [13], and in this article 95% of pregnancy-related PRES patients showed reversible MRI lesions, which support the view that pregnancy-related PRES has low mortality. In addition, Alhilali et al. believe that the rapid reversal of the rapid increase in blood pressure in patients with pregnancy-related PRES through urgent delivery may also be one of the reasons [8]. Although pregnancy is a protective factor for PRES death, it can still lead to miscarriage, so the related mechanisms still need to be further studied [14].

Previous studies have found that nephropathy is an independent risk factor for PRES [10], and hypertension caused by high fluid retention in patients with nephropathy may be known as the main reason. Many scholars believe that vasogenic edema caused by hypertension with dysregulation of cerebral blood flow and disruption of the blood-brain barrier are the pathological mechanism of PRES [15, 16]. This study found that kidney disease and the need for dialysis were also related to long-term death. After analysis, we found that 76.2% of patients with kidney disease were related to SLE, and 85.7% of patients who needed dialysis were also SLE patients. It is well known that the long-term prognosis of SLE with kidney disease is poor [17], which partly explains that PRES patients with kidney disease and dialysis had poor long-term prognosis.

In this article, we found for the first time that heart failure was an independent risk factor for long-term death in PRES. Our previous research has found that heart failure is an independent risk factor for PRES in SLE patients [10]. The main reasons are as follows: (1) heart failure mostly occurs in patients with severe renal dysfunction, accompanied by volume overload and high blood pressure, which explains that dysregulation of cerebral blood flow plays a role in the pathogenesis of PRES [15, 16] and (2) in addition, myocardial injury indicates that lupus is highly active, which may also imply that endothelial cell activation is involved in the pathogenesis of PRES. The 11 patients with heart failure in this article were also SLE patients, and most of the SLE patients with heart failure have multiple organ diseases, the treatment is more complicated, and the prognosis is worse [18]. Therefore, heart failure is not only a risk factor for PRES but also a risk factor for patient death.

For the first time, we confirmed that stroke was also a risk factor for long-term death through multivariate analysis. There are few studies on the prognosis of stroke (including hemorrhage and ischemia) and PRES. There is only one multifactor study on the 90-day prognosis of PRES patients and did not find that stroke is associated with prognosis [19], but a recent meta-analysis suggested that stroke suggests a poor prognosis [5]. This is the same result as our research, and our retrospective controlled study is more convincing. Stroke includes hemorrhagic and ischemic strokes. The incidence of hemorrhagic stroke and ischemic stroke (2.17%) in this article is relatively low. Previous literature recorded that the incidence of hemorrhagic stroke in PRES can reach 10–32%, which included subarachnoid hemorrhage, microhemorrhage, and cerebral parenchymal hemorrhage [20, 21]. There is a small amount of literature suggesting that hemorrhage is related to poor prognosis, but most of them are case studies and small-sample studies [4, 8], during which only Siebert et al. found subarachnoid can predict death in a study on risk factors for hospital death in PRES [22], and the results of this article suggested that hemorrhage was also significantly related to long-term death. According to reports, the incidence of ischemic stroke in PRES can also reach 11.26% [7]. Chen et al. believe that diffusion limitation is related to poor prognosis [5], but many literatures have not found that diffusion limitation is related to prognosis. In this paper, diffusion limitation was also not related to death but multivariate analysis found that ischemic stroke was related to prognosis. This also supports the view of some scholars from the side that severe cytotoxic edema is only related to the prognosis, but further research is needed in the future [5].

PRES is generally considered to be a relatively benign and reversible syndrome, and 75–90% of patients can recover completely, and clinical symptoms can last for 2–8 days [1, 2]. In this article, 90% of the patients fully recovered within a week. However, PRES may not be a completely reversible process. The study of Siebert et al. found that the hospital mortality rate of PRES can reach 11.2% [22], while in this article, the hospital mortality rate was relatively low, only 2.17%, and the all-cause mortality rate can reach 16% during the average 3-year follow-up. Legriel et al. found a follow-up mortality rate of 16% 90 days after discharge from the hospital in a follow-up study of severe PRES [19], which suggests that as survival time increases, the all-cause survival rate of PRES patients is basically stable, and found that more than 40% of patients had some degree of functional defect 90 days after PRES events. In this article, 4 patients were complicated with stroke, including 2 cases of subarachnoid hemorrhage and 2 cases of cerebral infarction, and they discharged from the hospital with obvious symptoms of neurological deficit. And at the end of the follow-up, 15.8% of patients still had different clinical symptoms. Therefore, timely diagnosis and reasonable treatment can improve mortality and are extremely important to prevent lasting neurological symptoms.

The higher value of this article is that it is a large-sample case-control study. Compared with most of the previous literatures which are case reports, the results of this study have higher credibility. However, our research still has some limitations. First, the retrospective design has certain flaws. The results depend on medical records and some clinical and laboratory data may be recorded inaccurately. Second, the possible results of a single-center study are somewhat restrictive. In addition, although the sample size of the death group in this article is relatively small, considering the relatively good prognosis of PRES, we believe that the sample size is sufficient.

As far as we know, this is the first large-scale controlled clinical study on the long-term prognosis of PRES. This study described the clinical characteristics, prognosis, and risk factors of death in patients with PRES. For the first time in a controlled study, we confirmed that stroke was an independent risk factor for long-term death, while pregnancy was a protective factor for death, and we firstly discovered that heart failure was also a risk factor for death. We found that PRES was not completely reversible, and the long-term prognosis was not very satisfactory. Therefore, when this syndrome is suspected clinically, the diagnosis should be confirmed as soon as possible and risk factors should be prevented in time to improve the long-term prognosis.

## Figures and Tables

**Table 1:** Basic characteristics and past history.

Variables	Death group	Survival group	*P* value
Sex (female/male)	3/13	11/65	0.665
Age (years)	23.44 ± 15.16	25.86 ± 9.79	0.421
History of hypertension (%)	31.3	17.1	0.195
History of lupus (%)	62.5	32.9	0.027
SBP at presentation (mmHg)^a^	156.56 ± 19.91	160.90 ± 26.38	0.546
Blood transfusion history within 1 month (%)	31.3	6.6	0.004
Pregnancy (%)	12.5	55.3	0.002
Operation history within 1 month (%)	12.5	1.3	0.077
Hypertension at the onset (%)	87.5	82.9	0.650

^a^The first SBP measured after an attack.

**Table 2:** Comparison of clinical manifestations.

Variables	Death group	Survival group	*P* value
Seizure (%)	75.0	72.4	0.830
Headache (%)	37.5	56.6	0.164
Visual impairment (%)	37.5	30.3	0.571
Coma (%)	18.8	19.7	0.928
Nephropathy (%)	75.0	39.5	0.010
Stroke (%)	18.8	1.3	0.016
Heart failure (%)	37.5	6.6	0.001

**Table 3:** Comparison of laboratory data.

Variables	Death group	Survival group	*P* value
Creatinine (mmol/L)	165.94 ± 193.84	96.23 ± 85.25	0.177
Urea nitrogen (*μ*mol/L)	12.37 ± 9.27	8.93 ± 12.17	0.290
WBC (×109)	12.07 ± 6.32	12.10 ± 6.24	0.985
Hgb (g/L)	102.5 ± 24.93	113.10 ± 23.18	0.104
PLT (×109)	147.19 ± 80.0	187.47 ± 128.68	0.233
NE (×109)	9.75 ± 5.79	10.06 ± 5.65	0.845
LY (×109)	1.44 ± 1.11	1.35 ± 0.90	0.739
LDH (U/L)	414.08 ± 134.31	633.82 ± 692.74	0.281
BNP (pg/mL)	8940.54 ± 10346.65	2654.20 ± 4483.27	0.053
LDL (mmol/L)	3.47 ± 1.57	3.27 ± 1.44	0.630
HDL (mmol/L)	1.38 ± 0.68	1.40 ± 0.64	0.958
Alb (g/L)	29.82 ± 8.52	30.71 ± 8.47	0.703
D-Dimer (mg/L)	3.011 ± 4.39	2.73 ± 6.62	0.183
Cholesterol (mmol/L)	5.67 ± 1.94	5.63 ± 1.82	0.932
Triglyceride (mmol/L)	3.06 ± 2.08	2.93 ± 1.79	0.800

Alb: albumin; BNP: brain natriuretic peptide; Hgb: hemoglobin; HDL: high-density lipoprotein; LY: lymphocyte; LDH: lactic dehydrogenase; LDL: low-density lipoprotein; NE: neutrophil; PLT: platelet; WBC: white blood cells.

**Table 4:** The lesion shown on MRI (*n*, %).

Lesion	Number	Percentage
Parietal lobe	87	94.6
Occipital lobe	78	84.8
Frontal lobe	72	78.3
Temporal lobe	61	66.3
Cerebellum	26	28.3
Lateral ventricles	12	13.0
Callosum	12	13.0
Basal ganglia	11	12.0
Thalamus	10	10.9
Brainstem	7	7.6
Typical MRI manifestations	74	80.4
DWI hyperintensity	63	68.5

**Table 5:** Treatment comparison.

Variables	Death group	Survival group	*P* value
Dialysis (%)	50.0	7.9	≤0.001
Tracheal intubation (%)	18.8	5.3	0.064
Antiepileptic therapy (%)	43.8	39.5	0.751
Antihypertensive drug (kinds)	2.06 ± 1.65	1.72 ± 1.68	0.464
The time of recovery from symptom (day)	4.63 ± 5.35	6.34 ± 9.71	0.496
Length of stay (day)	24.19 ± 18.19	18.08 ± 14.88	0.155

**Table 6:** The cause and the time of death.

Patient	Primary disease	Death cause	Death time after onset (month)
1	SLE	Heart failure	27
2	SLE	Progression	19
3	SLE	Progression	9
4	SLE	Progression	2
5	SLE	Heart failure	0.5
6	SLE	Lung infection	20
7	SLE	Progression	0.25
8	SLE	Progression	27
9	SLE	Progression	0.25
10	SLE	Lung infection	3
11	Cervical operation	Lung infection	2
12	Abdominal aneurysm operation	Operation again	53
13	Caesarean section(with Guillain-Barre's syndrome)	Lung infection	2
14	Hemophagocytic syndrome	Progression	2
15	Polycystic kidney	Lung infection	6
16	Hemophagocytic syndrome	Progression	4

**Table 7:** Risk factors for PRES.

Variables	Partial regression coefficient	*P* value	OR	95% CI
Pregnancy	2.077	0.017	7.978	1.446 to 44.006
Heart failure	−2.356	0.003	0.095	0.020 to 0.441
Stroke	−3.424	0.012	0.033	0.002 to 0.467

## Data Availability

The data used to support the findings of this study are available from the corresponding author upon request.
